# Etude de l´incidence des infections nosocomiales et facteurs de risque dans les maternités de la ville de Mbujimayi, République Démocratique du Congo

**DOI:** 10.11604/pamj.2021.38.95.15044

**Published:** 2021-01-28

**Authors:** Jean Christophe Bukasa, Pascal Muteba, André Kazadi, Didier Lepelletier, Félicien Ilunga, André Mutombo, Axel Ngoyi Kamanya, Angélique Bandimuna, Senghor Ngoyi Mbo, Wembonyama Stany

**Affiliations:** 1 Institut Supérieur des Techniques Médicales de Mbujimayi, Mbujimayi, République Démocratique du Congo,; 2 Laboratoire Emergent MiHAR, UFR Médecine, Université de Nantes, IRS2-Nantes Biotech, Nantes, France,; 3 Institut Supérieur des Techniques Médicales de Kinshasa, Kinshasa, République Démocratique du Congo,; 4 Université Officielle de Mbujimayi, Mbujimayi, République Démocratique du Congo,; 5 Institut Supérieur des Techniques Médicales de Kabinda, Kabinda, République Démocratique du Congo,; 6 Ecole de Santé Publique, Université de Lubumbashi, Lubumbashi, République Démocratique du Congo

**Keywords:** Incidence, infection nosocomiale, facteur de risque, maternité, Incidence, nosocomial infection, risk factor, maternity

## Abstract

**Introduction:**

cette étude vise à déterminer l´incidence des infections nosocomiales et les facteurs de risque chez les accouchées et les nouveau-nés dans les maternités de la ville de Mbujimayi en République Démocratique du Congo.

**Méthodes:**

il s´agit d´une étude descriptive longitudinale d´incidence et facteurs de risque des infections nosocomiales dans les 231 maternités, qui a été réalisée chez les sujets indemnes de la pathologie au départ qu´on devrait suivre en utilisant la collecte hebdomadaire des données pendant 6 mois. Les critères utilisés pour la collecte des données étaient ceux de l´Organisation Mondiale de la Santé (OMS) basés sur les définitions simplifiées pouvant être utiles pour certains établissements n´ayant pas accès à des techniques diagnostiques poussées.

**Résultats:**

l´incidence globale des infections nosocomiales chez les accouchées est de 24,8% et de 22,3% chez les nouveau-nés. Les facteurs de risque significatif d´infection nosocomiale au couple étaient les manœuvres instrumentales (p=0,005; OR=2,7; IC95% [1,3-5,4]), la césarienne faite en urgence (p=0,000; OR=2,3; IC95% [1,7-3,9]), l´utilisation d´un même flacon de collyre chez tous les bébés (p=0,004; OR=2,7; IC95% [1,4-5,5]) et l´élevage du prématuré hors couveuse (p=0,000; OR=2,61; IC95% [1,73-3,92]).

**Conclusion:**

la réalisation d'enquêtes d'incidence (ou à défaut de prévalence) régulières est indispensable pour évaluer les effets des actions d'information, de sensibilisation et de formation qui pourront être mises en place pour lutter contre les infections acquises à l'hôpital.

## Abstract (en)

**Introduction:**

this study aims to determine the incidence of nosocomial infections and the associated risk factors in new mothers and newborns at the maternity wards in the city of Mbujimayi, Democratic Republic of the Congo.

**Methods:**

we conducted a longitudinal descriptive study of the incidence and risk factors for nosocomial infections in patients without them at baseline in 231 maternity units. Data were collected on a weekly basis over a period of 6 months. Simplified WHO criteria were used for data collection. They are useful for hospitals that do not have advanced diagnostic techniques.

**Results:**

the overall incidence of nosocomial infections in new mothers was 24.8% and 22.3% in newborns. The significant risk factors for nosocomial infections in new mothers and newborns were instrumental maneuvers (p=0.005; OR=2.7; 95% CI [1.3-5.4]), emergency cesarean section (p=0.000; OR=2.3; 95% CI [1.7-3.9]), the use of the same eye-drop bottle in all babies (p=0.004; OR=2.7); 95% CI [1.4-5.5]) and preterm baby care outside an incubator (p=0.000; OR=2.61; 95% CI [1.73-3.92]).

**Conclusion:**

repeated incidence (or lack of prevalence) surveys are essential to assess the effects of information, awareness and training programs implemented to control hospital acquired infections.

## Introduction

Chaque année, le traitement et les soins dispensés à des centaines de millions des patients dans le monde ce sont compliqués par des infections contractées au cours de soins de santé. Dans certains pays en développement, la proportion de patients souffrant d´une infection résultant de soins de santé peut dépasser 25%. Certains patients se trouvent alors dans un état plus grave qu´il n´aurait été en situation normale. Certains doivent subir des hospitalisations prolongées, d´autres souffrent d´incapacités de longue durée et certains décèdent. Indépendamment du coût humain, les systèmes de soins de santé supportent une charge financière plus lourde [1].

Ces infections nosocomiales (IN) frappent environ 1 adulte sur 10 et 1 enfant sur 12 dans les hôpitaux de soins de courte durée. Elles représentent une importante préoccupation en matière de sécurité des patients en raison du risque pour les patients ainsi que des incidences économiques [2]. Les taux d´incidences cumulées retrouvés dans la littérature concernant les infections nosocomiales chez la mère varient de 0,5 à 65% selon le type d'accouchement; de 0,5 à 5% pour les accouchements par voie basse et de 1,6 à 65% pour les accouchements par césarienne. Chez les nouveau-nés, cette incidence est estimée entre 0,9 et 1,7% [3]. Les infections nosocomiales en maternité sont graves car responsables d'une morbidité maternelle et d'une surmortalité néonatale [4]. L'infection reste la deuxième cause de mortalité maternelle après l'hémorragie [5]. En Afrique, certaines études déjà menées sur les infections nosocomiales en maternité ont montré que la prévalence de ces infections, varie entre 10 et 60% [2]. Ces infections nosocomiales représentent en Afrique, la troisième cause de mortalité maternelle, la deuxième cause de mortalité néonatale précoce, et la première cause de morbidité postopératoire.

Toutes ces réalités des infections nosocomiales en maternité n´épargnent pas les maternités ainsi que les unités de néonatologie de la ville de Mbujimayi et elles peuvent toucher à la fois la mère et l'enfant. C´est dans ce cadre que nous trouvons qu´il était crucial de dégager l´incidence des infections nosocomiales en maternité et ressortir les facteurs de risque chez les accouchées et les nouveau-nés afin de contribuer ainsi à la mise en place d´un mécanisme de prévention continue, de surveillance des infections nosocomiales à travers le Réseau d´Alerte, de Surveillance et Prévention des Infections Nosocomiales (RASPIN) que nous avons créé à la clinique de l´Institut Supérieur de Techniques Médicales de Mbujimayi, comme contribution de cette étude à la réduction tant soit peu de ces infections dans les maternités de la ville de Mbujimayi.

## Méthodes

Il s´agit d´une étude descriptive longitudinale d´incidence et facteurs de risque des infections nosocomiales dans les maternités, qui a été réalisée chez les sujets indemnes de la pathologie au départ qu´on devrait suivre dans les groupes exposés et non exposé en utilisant la collecte hebdomadaire des données pendant 6 mois.

La population concernée était composée de l´ensemble des accouchées et nouveau-nés ayant séjourné plus de 48 heures dans chaque maternité. L´échantillon est du type accidentel ou de convenance. Il était constitué de 6006 parturientes ayant accouchées par voie basse et/ou par césarienne et de 6068 nouveaux nés (à terme et prématurés), parmi lesquels nous avions trouvé 31 jumeaux.

En rapport avec le déroulement de l´enquête, le recueil de données s´est fait au moyen d´une fiche standardisée, remplie par un enquêteur formé, un étudiant de troisième ou de deuxième année de graduat en sciences infirmières, supervisé par une accoucheuse de la maternité. Dans l'ensemble, les étudiants de troisième graduat au nombre de 231 ont effectué ces enquêtes pendant 3 mois soit du 1^er^ novembre 2016 au 1^er^ février 2017 et chacun devrait passer dans une maternité bien lui désignée, proche de son domicile et avait 2 jours chaque semaine (lundi et jeudi) de passage à la maternité pour collecter les données. Ils ont été remplacés pour la suite de l´enquête qui avait encore duré 3 mois soit du 2 février 2017 au 2 mai 2017 par les étudiants de deuxième graduat. La durée journalière de l´enquête pour chaque maternité était en moyenne de 4 heures pour 6 à 7 accouchées et nouveau-nés enquêtées. Ce qui permettait aux enquêteurs de mettre 30 minutes pour chaque couple mère-enfant afin de déterminer combien d´accouchées / nouveau-nés avaient développé une infection nosocomiale et en dégager les facteurs de risque.

Pendant la collecte des données, était considéré comme une infection nosocomiale toute infection qui s´est manifesté chez le couple mère-enfant ou soit l´un des deux, 48 heures après admission à la maternité et qui n´était ni présente à l´entrée, ni en incubation. Les critères utilisés étaient ceux de l´OMS basés sur les définitions simplifiées pouvant être utiles pour certains établissements n´ayant pas accès à des techniques diagnostiques poussées, utilisables aux fins d´enquête par les établissements ne disposant que d´un accès limité à des techniques de diagnostic sophistiquées [6]. Aussi les facteurs de risques significatifs d´une infection nosocomiale chez ce couple, sur lesquels les enquêteurs devraient se pencher étaient: les manœuvres instrumentales, la césarienne faite en urgence, l´utilisation d´un même flacon de collyre chez tous les bébés et l´élevage du prématuré hors couveuse. Les examens de laboratoire à caractère bactériologique ont été insuffisamment demandés et réalisé dans la plupart de maternités. Pour se faire une idée sur l´écologie bactérienne responsable des infections nosocomiales dans les maternités de la ville de Mbujimayi, quelques prélèvements de 30 échantillons d´urines, de sang, des écouvillons de l´endocol, des sécrétions oculaires, de pus et sérosités pour chacune des catégories du couple mère-enfant selon le cas ont été réalisés. Ces échantillons ont été analysés au laboratoire de l´Institut Supérieur des Techniques Médicales de Mbujimayi moyennant les milieux de culture, don de la firme allemande « *PHYWE SYSTEM* ». Les données ainsi récoltées ont été enregistrées sur un masque de saisie Excel 2013 et importées sur épi info version 3.5 pour analyse descriptive et bivariée.

## Résultats

L´incidence globale des infections nosocomiales chez les accouchées dans les maternités de la ville de Mbujimayi est de 24,8% de cas, de 24,6% (accouchées par voie basse) et de 30,3% chez les accouchées par césarienne. La comparaison de leur taux respectifs par les 2 voies d´accouchements et par rapport aux types des structures a confirmé la relation d´une manière significative (p< 0,05) (Tableau 1).

**Tableau 1:** incidence des infections nosocomiales chez les accouchées et les nouveau-nés selon les types de structures

	LES ACCOUCHEES						LES NOUVEAU-NES						
Types de structures	Nbre d´accouchement	Cas et Taux (%) d´IN chez les mères	Cas et Taux (%) d´IN chez les accouchées par voie basse	Cas et Taux (%) d´IN chez les accouchées par césarienne	X2	p	Nbre de naissances	Cas et Taux (%) d´IN chez les N.N	Cas et Taux (%) d´IN chez les N.N à terme	Cas et Taux (%) d´IN chez les N.N prématurés.	X2	p
(5) Etatique et (45) conventionnel	1074	159 (14,8)	110/913(12,0%)	49/161(30,4)	329,84	0,000	1106	168 (15,2)	123/924(13,3)	45/182(24,7)	225,95	0,000
(181) Privé	4932	1329 (26,9)	1318/4895(26,9)	11/37(29,7)			4962	1185 (23,9)	1170/4907(23,8)	15/52 (28,8)		
Total	**6006**	**1488 (24,8)**	**1428/5808(24,6)**	**60/198(30,3)**			**6068**	**1353 (22,3)**	**1290/5831(22,1)**	**57/234(24,3)**		

L´incidence globale des infections nosocomiales chez les nouveau-nés dans les maternités de la ville de Mbujimayi est de 22,3% de cas. Le taux d´infections nosocomiales chez les nouveau-nés à terme est de 22,2% et elle est de 25,5% chez les prématurés. La comparaison de leur taux d´infections nosocomiales par rapport aux types des structures a confirmé la relation de manière significative (p< 0,05) (Tableau 2).

**Tableau 2:** incidence des infections nosocomiales selon les caractéristiques sociodémographiques des accouchées

Caractéristiques	Effectif n=1488	
**Par voie basse n= 1428 (%)**	**Par césarienne n=60 (%)**
**Age de l´accouché**		
<30 ans	533 (37,3)	48 (80)
>30 ans	895 (62,7)	12 (20)
**Etat civil**		
Mariée	1248 (87,9)	56 (93,3)
Autres	180 (12,1)	4 (6,7)
**Gestite**		
Primigeste	583 (40,5)	24 (40)
Multigeste	845 (59,5)	36 (60)
**Parité**		
Primipares	579 (40,6)	22 (36,6)
Multipares	849 (59,4)	38 (63,4)
**Niveau d´instruction**		
Sans niveau	371 (25,9)	15 (25)
Primaire	570 (40,0)	27 (45)
Secondaire	367 (25,7)	13 (22)
Universitaire	120 (8,4)	5 (8)
**Occupation**		
Sans occupation	385 (27)	16 (26,7)
Ménagère	488 (34,2)	19 (31,7)
Vendeuse	546 (38,2)	23 (38,3)
Fonctionnaire de l’état	9 (0,6)	2 (3,3)
**Durée moyenne de séjour (DMS)**		
<10 jours	387 (27,1)	15 (25)
>10 jours	1041 (72,9)	45 (75)

Les accouchées âgées de plus ou moins égal à 30 ans représentent 64,3% des cas. Par rapport à l´état civil, 87,9% d´entre elles sont mariées; la majorité étaient des multipares et multigestes avec respectivement 60,9% et 61%. Quarante et un virgule sept pourcent des accouchées avaient un niveau primaire, 36,8% étaient des vendeuses et 73,9% des accouchées ont eu une durée moyenne de séjour supérieur à 4 jours (Tableau 3).

**Tableau 3:** incidence des infections nosocomiales selon les caractéristiques sociodémographiques des nouveau-nés

Caractéristiques	Effectif n=1353	
**A terme n=1290 (%)**	**Prématuré n=57 (%)**
**Age du nouveau-né**		
3-5 jours	450 (34,9)	20 (35,1)
6 jours et plus	840 (65,1)	37 (64,9)
**Sexe**		
Masculin	564 (43,8)	25 (43,8)
Féminin	726 (56,2)	32 (56,2)
**Poids de naissance**		
<2500 grs	60 (4,7)	3 (5,3)
2500 grs et plus	1230 (95,3)	54 (94,7)
**Durée moyenne de séjour (DMS)**		
<6 jours	184 (14,3)	15 (8,1)
>6 jours	1106 (85,7)	45 (91,9)

**Figure 1:**
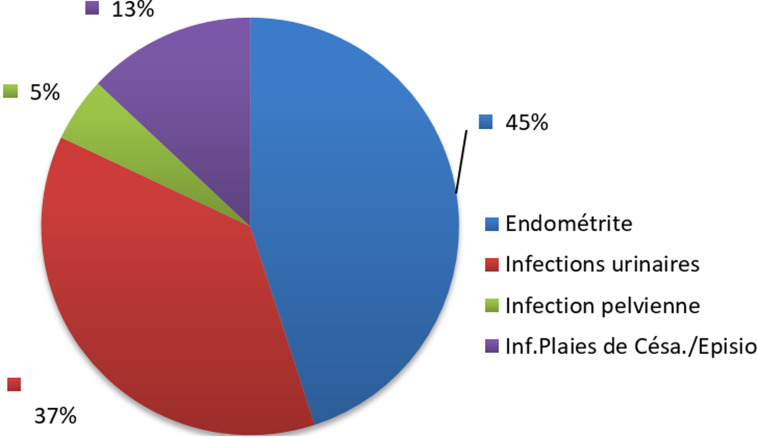
incidence selon les types d’infections nosocomiales chez les accouchées

**Figure 2:**
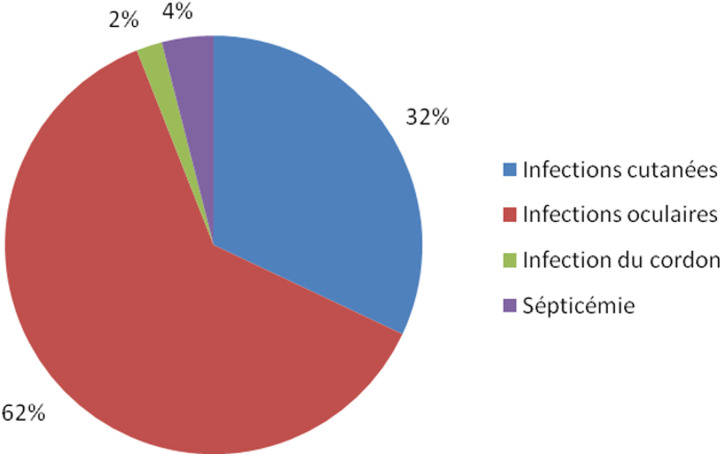
incidence selon les types d’infections nosocomiales chez les nouveau-nés

Les nouveau-nés dans la tranche d´âge de 6 jours et plus étaient plus touchés par les infections nosocomiales dans 66,7% de cas. Les nouveau-nés de sexe féminin étaient majoritaires avec 58,3% de cas. En rapport avec le poids à la naissance, les nouveaux nés avec un poids inférieur à 2500 grammes étaient en surnombre avec 95,6% des cas (Tableau 4). Au cours de cette étude, en rapport avec les types d´infections, 45% des accouchées avaient développé l´endométrite. Trente-sept pourcent avaient développé une infection des voies urinaires, 13% une infection des plaies de césarienne et/ou d´épisiotomie et 5% d´accouchées avaient développé une infection pelvienne (Figure 1). Chez les nouveaux nés, 62% avaient développé une infection oculaire, 31% avaient développé une infection cutanée, 4% un sepsis et 2% une infection du cordon ombilical (Figure 2).

**Tableau 4:** quelques germes des infections nosocomiales isolés chez les accouchées et les nouveau-nés

	Les accouchées			Les nouveaux nés			
**Types d´IN germes isolés**	Endométrite / Infection pelvienne n=14	Infection des voies urinaires n=11	Infection des plaies de césarienne / épisiotomie N=5				
*Enterobacter pyrogène*	5 (35,7%)	3 (27,3%)	1 (20%)				
*Staphylococcus aureus*	7 (50%)	1 (9,1%)	2 (40%)				
*Proteus mirabilis*	2 (14,3%)	-	1 (20%)				
*Staphylococcus albus*	-	1 (9,1%)	-				
*Escherichia coli*	-	6 (54,5%)	-				
*Proteus vulgaris*	-	-	1 (20%)				
**Types d´IN germes isolés**				Infection cutanée n=9	Infection oculaire n=19	Infection du cordon n=1	Sepsis néonatal n=1
*Staphylococcus epidermidis*				7 (77,8%)	_	_	_
*Staphylococcus aureus*				2 (22,2%)	4 (21,1%)	_	1 (100%)
*Proteus mirabilis*				_	_	1 (100%)	_
*Staphylococcus albus*				_	13 (68,4%)	_	_
Staphylocoques à coagulase négative				_	2 (10,5%)	_	_

L´écologie bactérienne liée aux infections nosocomiales trouvée dans notre étude au travers quelques prélèvements se présente de la manière suivante: dans le cas d´endométrite, les germes isolés étaient le *Staphylococcus aureus* (50%), *Enterobacter pyrogène* (35,7%) et *Proteus mirabilis* (14,3%). Pour les infections des voies urinaires, on avait plus isolé *Escherichia coli* (54,5%), *Enterobacter pyrogène* (27,3%), *Staphylococcus albus* et *Staphylococcus aureus* respectivement (9,1%). Dans les infections des plaies de césarienne/épisiotomie, c´était plus le *Staphylococcus aureus* (40%), le *Proteus mirabilis, Proteus vulgaris* et *Enterobacter pyrogène* (27,3%) (Tableau 5). Pour le nouveau-né, les *Staphylococcus epidermidis* (77,8%) et *Staphylococcus aureus* (22,2%) ont été isolés dans infections cutanées, le *Staphylococcus albus* (68,4%), le *Staphylococcus aureus* (21,1%), les staphylocoques à coagulase négative (10,5%) ont été plus isolé dans les infections oculaires. Le *Proteus mirabilis* et le *Staphylococcus aureus* ont été les seuls germes isolés respectivement dans les infections du cordon et le sepsis néonatal (Tableau 5).

**Tableau 5:** analyse multi variée des facteurs de risque des infections nosocomiales chez les accouchées et les nouveau-nés

	Cas et Taux d´inf. (%) (n/N)	OR (IC =95%)	p
**Manœuvres instrumentales**	(132/3087) 4,3%	2,7 ; IC95% [1,3-5,4]	0,005
**Césarienne d´urgence**	(169/196) 85,4%	2,3 ; IC95% [1,7-3,9]	0,000
**Utilisation d´un même flacon de collyre chez tous les bébés**	(512/3762) 13,6%	2,7 ; IC95% [1,4-5,5]	0,004
**Elevage du prématuré hors couveuse**	(98/234) 41,8%	2,61 ; IC95% [1,73-3,92]	0,000

Par rapport aux facteurs de risque des infections nosocomiales chez les accouchées et les nouveaux nés, les variables d´exposition aux différents types d´infections nosocomiales chez les accouchées par voie basse ou par césarienne, chez les nouveaux nés à terme et chez les prématurés ayant un p<0,20 ont été intégrées dans l´analyse multivariée. A l´issue de cette analyse, les résultats suivants ont été trouvé: pour les accouchées par voie basse: il s´avère que seules les manœuvres instrumentales sont un facteur de risque significatif d´infection nosocomiale (endométrite) de la mère (p=0,005; OR=2,7; IC95% [1,3-5,4]); pour les accouchées par césarienne: seule la césarienne faite en urgence a été un facteur de risque significatif d´ISO de la mère (p=0,000; OR=2,3; IC95% [1,7-3,9]); pour le nouveau-né à terme, seule l´utilisation d´un même flacon de collyre chez tous les bébés reste un facteur de risque significatif d´infection oculaire chez le nouveau-né (p=0,004; OR=2,7; IC95% [1,4-5,5]); pour le nouveau-né prématuré, seule l´élevage du prématuré hors couveuse a été un facteur de risque significatif de l´infection cutanée chez le nouveau-né (p=0,000; OR=2,61; IC95% [1,73-3,92] (Tableau 5)).

## Discussion

Cette étude vise à déterminer l´incidence des infections nosocomiales et les facteurs de risque chez les accouchées et les nouveau-nés dans les maternités de la ville de Mbujimayi en République Démocratique du Congo.

Chez l´accouchée et le nouveau-né: cette étude a montré que l´incidence globale des infections nosocomiales chez les accouchées dans les maternités de la ville de Mbujimayi est de 24,8% de cas; par voie basse elle est de 24,6% et est de 30,3% chez les accouchées par césarienne. Tandis que les nouveaux nés présentent une incidence globale de 22,3% de cas; chez les nouveaux nés à terme elle est de 22,2% et de 25,5% chez les prématurés. Nos résultats sont de loin supérieurs à Malavaud S *et al*. [5] et Vincent-Boulétreau A *et al*. [7] qui avaient trouvé que les taux d´infections nosocomiales chez les femmes accouchant par césarienne et par voie basse avaient respectivement diminué de 7,8 à 4,3% (p ≪ 0,001) et de 2,2 à 0,9% (p ≪ 0,001) et à ceux de Sécher I *et al*. [8] qui avaient rapporté à leur tour une incidence des infections nosocomiales en 2005 et en 2008 de 3,5% et 6,1% pour les accouchements par voie basse, 15,4% et 13,2% pour les césariennes, 2,3% et 0,9% pour les nouveau-nés. L´incidence des infections du site opératoire était de 3,6% en 2008 dans l´enquête «ISO» et dans l´enquête Mater [3]. Néanmoins en ce qui concerne les types d´infections, nos résultats approchent ceux de Saizonou J *et al*. [9] qui avaient renseigné une incidence des infections du per-partum de 5,9 pour 100 accouchements (110 / 1875); les types d'infections les plus incriminés étaient les endométrites (27,3%), les chorioamniotites (18,2%), les infections du site opératoire (12,7%) et les infections urinaires (2,7%). En rapport avec ces résultats Chabni N *et al*. [4] affirment également que les principales infections rencontrées chez la mère sont les endométrites, les infections urinaires, les infections du site opératoire, les infections du sein et chez les nouveau-nés, les infections cutanées et les infections oculaires sont les plus fréquentes.

Les caractéristiques sociodémographiques des accouchées et des nouveau-nés: Cette étude révèle que les accouchées d´âge supérieur à 30 ans représentaient 64,3% des cas. Par rapport à l´état civil, 87,9% d´entre elles, étaient des mariées, la majorité étaient multipares et multigestes avec respectivement 60,9% et 61%. Quarante et un virgule sept pourcent des accouchées avaient un niveau primaire, 36,8% étaient des vendeuses et 73,9% des accouchées ont eu une durée moyenne de séjour hospitalier supérieur à 4 jours. Le délai moyen de survenue des épisodes infectieux des plaies de césarienne est de 6 ± 1,23 jours d´hospitalisation. Nos résultats corroborent ceux de Saizonou J *et al*. [9] qui avaient constaté que la plupart de ces mères étaient âgées de 19 à 34 ans; tranche d´âge les plus exposées aux activités de maternité, mariées en grande majorité et donc ayant une charge de travail ménagère élevée. Elles étaient dans leur majorité non scolarisées ou de niveau primaire, ayant donc des connaissances et des pratiques limitées et peu favorables à l'application des règles d'hygiène de base. Tandis que les nouveau-nés âgés de 6 jours et plus étaient plus touchés par les infections nosocomiales dans 66,7% de cas, ceux de sexe féminin représentaient 58,3% de cas et les nouveau-nés hypotrophiques ou les faibles poids de naissance (FPN) (poids inférieur à 2500 grs) avec 95,6% des cas. Ces résultats sont en désaccord avec ceux rapportés par Chabni-Settouti N *et al*. [10], qui avaient trouvé dans leurs études que les filles représentaient 42% (n=1657) et les garçons, 58% (n=2298) avec sex ratio de 1,39. L´âge moyen à l´admission est de 3,05 ± 0,15 j (1-30). La durée moyenne d´hospitalisation est de 4,7 ± 0,16 jours.

Types d´infections nosocomiales chez les accouchées et les nouveaux nés. Au cours de cette étude, 45% des accouchées avaient développé l´endométrite. Or dans l´étude multicentrique menée par Jacobsson B *et al*. [11], un taux d'incidence supérieur d'endométrite de 50% a été trouvé et elle était 3 à 4 fois plus fréquente après la césarienne qu´après l'accouchement par voie basse. Trente-sept pourcent avaient développé une infection des voies urinaires, 13% une infection des plaies de césarienne et/ou d´épisiotomie et 5% d´accouchées avaient développé une infection pelvienne. Ces résultats diffèrent en partie de ceux de Mbutshu Lukuke H [12] qui, dans son étude avait trouvé une incidence de 14,4% pour les infections urinaires et 9,9% pour les ISO. Chez les nouveaux nés, 62% avaient développé une infection oculaire, ce qui n´est pas superposable aux résultats d´Olivier M [13], qui avait trouvé dans son étude que les infections oculaires représentaient 82% des infections. Leur incidence était de 0,3% (9/2986). 31% avaient développé une infection cutanée, 4% un sepsis et 2% une infection du cordon ombilical, alors que Chabni-Settouti N *et al*. [10], avaient trouvé que les sepsis représentaient les infections prédominantes (76%).

Quelques germes isolés selon les types d´infections nosocomiales chez l´accouchée et le nouveau-né. L´écologie bactérienne liée aux infections nosocomiales trouvée dans notre étude au travers quelques prélèvements se présente de la manière suivante: dans le cas d´endométrite, les germes plus isolés étaient le *Staphylococcus aureus* (50%). Pour le nouveau-né, les *Staphylococcus epidermidis* (77,8%) a été plus isolé dans infections cutanées, le *Staphylococcus albus* (68,4%), le *Staphylococcus aureus* (21,1%). Le *Proteus mirabilis* et le *Staphylococcus aureus* ont été les seuls germes isolés respectivement dans les infections du cordon et le sepsis néonatal.

Cette écologie est similaire à celle décrite dans d´autres enquêtes européennes à l´exception du *Shigella* spp. selon Kouchner B [14] et dans une autre enquête menée à Lubumbashi en RDC par Mbutshu Lukuke H [11]. Les facteurs de risque des infections nosocomiales chez les accouchées et les nouveaux nés. Les variables d´exposition aux différents types d´infections nosocomiales chez les accouchées par voie basse ou par césarienne, chez les nouveaux nés à terme et chez les prématurés ayant un p < 0,05 ont été intégrées dans l´analyse multivariée. A l´issue de cette analyse, les résultats suivants ont été trouvé: Pour les accouchées par voie basse: il s´avère que seules les manœuvres instrumentaux sont un facteur de risque significatif d´infection nosocomiale (endométrite) de la mère (p=0,005; OR=2,7; IC95% [1,3-5,4]); pour les accouchées par césarienne: seule la césarienne faite en urgence a été un facteur de risque significatif d´ISO de la mère (p=0,000; OR=2,3; IC95% [1,7-3,9]); pour le nouveau-né à terme, seule l´utilisation d´un même flacon de collyre chez tous les bébés reste un facteur de risque significatif d´infection oculaire chez le nouveau-né (p=0,004; OR=2,7; IC95% [1,4-5,5]; Pour le nouveau-né prématuré, seule l´élevage du prématuré hors couveuse a été un facteur de risque significatif de l´infection cutanée chez le nouveau-né (p=0,000; OR=2,61; IC95% [1,73-3,92].

Ces résultats sont conformes à plusieurs études menées en Europe, en Afrique voir en République Démocratique du Congo qui confirment qu´un grand nombre (7 à 8) de touchers vaginaux favorise la survenue d'une chorio-amniotite chez une femme avec une rupture prématurée des membranes selon Lemarie C [15]. Ce facteur de risque a concerné près du tiers de nos mères. L'extraction fœtale par voie haute expose tout particulièrement la mère aux infections nosocomiales, la césarienne multipliant le risque par 8 [16]. L'infection urinaire est la complication la plus fréquente après cette intervention. Viennent ensuite l'infection de paroi, qui serait très opérateur dépendant, puis les endométrites qui seraient multipliées par 3 à 4. Celles-ci seraient également plus fréquentes dans les grossesses multiples, les ruptures prématurées de la poche des eaux supérieures à 12 heures et après corticothérapie [17].

## Conclusion

Cette étude nous a permis d´aboutir aux résultats selon lesquelles, l´incidence globale des infections nosocomiales chez les accouchées était de 24,8%% et de 22,3% chez les nouveau-nés. Les types d'infections les plus incriminés dans le couple mère-enfants étaient les endométrites (45%), les infections des plaies de césarienne et/ou d´épisiotomie (13%), les infections urinaires (37%), les infections pelviennes (5%), l´infection du cordon ombilical (2%), le sepsis néonatal (4%), les infections cutanées (32%), et les infections oculaires (62%). Les facteurs de risque significatif d´infection nosocomiale au couple étaient les manœuvres instrumentales (p=0,005; OR=2,7; IC95% [1,3-5,4]), la césarienne faite en urgence (p=0,000; OR=2,3; IC95% [1,7-3,9]), l´utilisation d´un même flacon de collyre chez tous les bébés (p=0,004; OR=2,7; IC95% [1,4-5,5] et l´élevage du prématuré hors couveuse (p=0,000; OR=2,61; IC95% [1,73-3,92]. Partant de ces résultats et étant donné que les services de gynécologie-obstétrique sont, comme les autres unités de soins, exposés aux IN, les actions de prévention et de sensibilisation, contrôlées régulièrement par des enquêtes d'incidences, s'avèrent indispensables pour lutter contre cette pathologie. La simple surveillance diminue de façon importante les infections nosocomiales. Nous espérons que si cette expérience d'information, de sensibilisation et de la formation des personnels ainsi que la surveillance par une enquête d'incidence de 3 mois tous les 2 ans est mis en œuvre, elle pourra diminuer le taux d´IN dans les maternités de la ville de Mbujimayi dans les années à venir. Ce qui a valu la création d´un Réseau d´Alerte, de Surveillance et Prévention des Infections Nosocomiales (RASPIN) rattaché à la clinique de l´Institut Supérieur des Techniques Médicales de Mbujimayi, comme contribution de la présente étude à la science. Cette lutte permanente sera essentielle pour les patients, elle contribuera à la qualité des soins prodigués et pourra concourir à la réduction des risques médico-légaux et à la mortalité du couple mère-enfant.

### Etat des connaissances sur le sujet

- L´incidence et les facteurs de risque des infections nosocomiales chez l´accouchée et le nouveau-né sont connus pour les maternités des pays d´Europe;
- L´incidence et les facteurs de risque des infections nosocomiales chez l´accouchée et le nouveau-né sont également connue pour les maternités des USA;
- Les infections nosocomiales constituent la deuxième cause de mortalité maternelle après les hémorragies du post partum chez l´accouchée et l´une des premières causes de décès du nouveau-né.

### Contribution de notre étude à la connaissance

- Une étude d´incidence et les facteurs de risque est menée spécifiquement pour les infections fois dans les maternités de la ville de Mbujimayi car l´étude de Mbutshu Lukuke H s´était penché sur les autres services des hôpitaux de Lubumbashi et avait trouvé une incidence de 14,4% pour les infections urinaires et 9,9% pour les ISO;
- L´incidence et les facteurs de risque des infections nosocomiales chez l´accouchée dans les maternités de la ville de Mbujimayi sont maintenant connue. Cette incidence globale des infections nosocomiales chez les accouchées est de 24,8% et les facteurs de risque significatif d´infection nosocomiale chez les accouchées étaient les manœuvres instrumentales (p=0,005; OR=2,7; IC95% [1,3-5,4]) et la césarienne faite en urgence (p=0,000; OR=2,3; IC95% [1,7-3,9]);
- L´incidence et les facteurs de risque des infections nosocomiales chez le nouveau-né dans les maternités de la ville de Mbujimayi sont maintenant connue. L´incidence globale des infections nosocomiales chez les chez les nouveau-nés est de 22,3% et les facteurs de risque sont l´utilisation d´un même flacon de collyre chez tous les bébés (p=0,004; OR=2,7; IC95% [1,4-5,5] et l´élevage du prématuré hors couveuse (p=0,000; OR=2,61; IC95% [1,73-3,92].

## Remerciements

Nous remercions tous les auteurs qui ont contribué à la lecture et correction de cet article sur le plan du fond et de la forme ainsi que pour l´analyse des données au moyen de l´épi-info. Nous remercions également toutes les accoucheuses et sages-femmes de différentes maternités de la ville de Mbujimayi pour avoir supervisé durant les 6 mois l´enquête d´incidence et facteurs de risque qui avait abouti à la rédaction de cet article. Nos remerciements vont également tout droit à l´endroit des enquêteurs tous étudiants stagiaires de G3 et G2 Sciences infirmières à l´Institut Supérieur de Techniques Médicales de Mbujimayi pour avoir participé d´une manière bénévole durant 6 mois à cette enquête d´incidence et facteurs de risque.
